# Advances in Natural and Bio-Inspired Nanoparticles for the Treatment of Cardiovascular Diseases

**DOI:** 10.3390/nano13233015

**Published:** 2023-11-24

**Authors:** Mariana Varna, Manuela Calin, Ille C. Gebeshuber

**Affiliations:** 1 Paris-Saclay University, Institut Galien Paris-Saclay, CNRS UMR 8612, Pole Biologie-Pharmacie-Chimie, Bâtiment Henri Moissan, 6 Rue d’Arsonval, 91400 Orsay, France; 2 Medical and Pharmaceutical Bionanotechnologies Laboratory, Institute of Cellular Biology and Pathology “Nicolae Simionescu” of the Romanian Academy, 8 B.P. Hasdeu, 050568 Bucharest, Romania; 3 Institute of Applied Physics, TU Wien, Wiedner Hauptstrasse 8-10/134, 1040 Vienna, Austria

Cardiovascular diseases (CVD) is a general term for disorders affecting the heart or blood vessels and represent a major cause of disability and death worldwide [1]. There are different kinds of CVD, which are, among others, coronary heart disease, stroke, transient ischemic attack (TIA), peripheral arterial disease, and aortic disease. Coronary heart disease appears when the oxygenated blood flow to the heart muscle is reduced or blocked, leading to angina, heart attack, or heart failure. A stroke is induced by the cut-off of the blood supplying the brain, whereas, in a TIA, the blood flow to the brain is only temporarily interrupted. Peripheral arterial disease is induced when flow blockage appears in the arteries of the limbs. While these three CVDs can be induced by the presence of a thrombus, in aortic diseases, the walls of the aorta are weakened and swell outwards with an increased risk of breaking [2].

A major underlying cause of clinical manifestation of CVD, such as myocardial infarction, stroke, and peripheral arterial disease, is atherosclerosis, a systemic disease characterized by lipid deposition and a chronic inflammatory process in the arterial walls. The risk factors leading to atherosclerosis are generally known, such as smoking, high blood pressure, high cholesterol, diabetes, and obesity to cite only a few. In time, the complications of atherosclerosis lead to unstable plaque, which is prone to rupture and thrombus formation. Apart from reducing risk factors, there are some therapeutic strategies to prevent these diseases or to treat them once triggered. Thus, a large number of therapeutic options have been developed for the management of cardiovascular diseases. However, despite substantial improvements achieved in the treatment, there is still an essential need for drug innovation. 

This is in part due to the complexity of diseases, which involves an excessive response from the organism and is eventually deleterious to the organ itself. One example is inflammation that occurs after myocardial ischemia/reperfusion or after a stroke. Reperfusion is the restoration of blood flow to an organ or tissue after having been blocked. It is essential for reducing acute mortality after myocardial ischemia but can also be a double-edged sword. Hence, reperfusion triggers a strong, uncontrolled inflammatory reaction that is heavily involved in subsequent complications, such as the onset of heart failure. The whole-body distribution of an administered drug, and its rapid clearance before reaching the zone of interest, represent a major limitation in current therapies. 

For a few decades, nanomedicine has emerged as a candidate to either improve therapeutic efficacy or to achieve a diagnosis purpose. As stated by P. Couvreur, the term nanomedicine refers to “a biologically active molecule formulated as nanoparticles to improve or control the pharmacokinetics and the biodistribution of the active principle in order to ameliorate the therapeutic efficacy” [3]. 

The field of nanomedicine has continued to improve since its initial discovery. Thus, the very first nanomedicines demonstrated the proof of concept that it is possible to protect an active ingredient from rapid metabolization in the body. This was followed by the concept of “stealth” nanomedicine, with PEGylated nanoparticles [4], which enable prolonged circulation in the bloodstream. A new stage in the complexity of nanomedicines is emerging with the development of targeting nanoparticles (i.e., decoration of the nanoparticle surface with specific ligands for precise drug delivery) or with the development of stimuli-responsive nanomedicines. Made of a multitude of materials, biodegradable or non-biodegradable, organic or inorganic, nanomedicines had their first clinical applications in the treatment of cancer [5]. Since then, further applications of nanoparticle-based strategies have emerged, such as for infectious, neurological, or inflammation-associated diseases [6,7,8]. 

In the cardiovascular field, special attention is given to bio-inspired and biomimetic entities as a novel drug delivery platform to enhance drug biocompatibility, ameliorate pharmacokinetics, and avoid the rapid clearance of the drug. Most biomimetic and bio-inspired nanosystems are cell derived (e.g., erythrocytes and platelets): extracellular vesicles, viruses, bacteria, proteins (e.g., albumin), synthetic HDL, or squalene based, to cite only a few examples. For review, see, e.g., Godin et al. [9], Deng et al. [10], and Mohamed et al. [11].

Extracellular vesicles (EVs) have been widely used in the CVD field. EVs are an endogenous and highly heterogeneous kind of nanosystem, 30–160 nm in size, composed of a phospholipid bilayer with complex contents rich in proteins, lipids, and nucleic acids. Furthermore, efforts have focused on the development of so-called “mimetic” HDL nanoparticles, composed, for example, of a poly(lactic-co-glycolic) acid (PLGA) core and a coating of lipids and ApoA, a main protein of HDL. As erythrocytes are components of the blood, erythrocytes-derived NPs were developed to deliver either active principles or for imaging purposes. 

Due to their excellent biocompatibility and biodegradability, the use of biomimetic and bio-inspired nanomedicines would provide a novel and promising strategy for health care. There is no doubt that interdisciplinary collaborations between different fields will advance the knowledge in this research area of nanomedicines.

The Special Issue “Advances in Natural and Bio-Inspired Nanoparticles for the Treatment of Cardiovascular Diseases” of the MDPI journal *Nanomaterials* aims to present comprehensive research outlining the progress of applying bio-inspired nano-systems to improve therapy or diagnosis in the cardiovascular field. The types of manuscripts accepted are full papers, short communications, reviews, points of view, and methodological articles.

The published manuscript “A Review on the Applications of Natural Biodegradable Nano Polymers in Cardiac Tissue Engineering” [12] by the authors from Italy and Serbia is an example of the high interdisciplinarity of the field: mathematics, materials, biosystems, and nanotechnology researchers working together synergistically. The review article discusses the significance and applications of natural biodegradable nano-polymers in cardiac tissue engineering and emphasizes the importance of tissue engineering in addressing cardiac-related issues and the potential of nano polymers in this domain. The methods reviewed include electrospinning and 3D bioprinting. The biomaterials reviewed include collagen, gelatin, and chitosan. The techniques reviewed include UV-Assisted 3D Bioprinting and the use of cyclic RGDfK peptides to improve the survival of transplanted cells in damaged myocardium. RGDfK peptides are of interest in medical research because of their ability to interact with cells, especially in areas such as cancer therapy and tissue engineering [13]. RGDfK peptides can be used to target drugs to specific cells or to influence how cells interact with materials in the body. The review explores the potential applications of the nano-polymers in cardiac repair, regeneration, and tissue engineering and discusses the use of different scaffolds, such as ones made from collagen, gelatin, alginate, and chitosan, for cardiac tissue engineering. The authors underline the significance of natural and bio-inspired biodegradable nano-polymers in cardiac tissue engineering and the potentials they hold for the future of cardiac treatments and interventions.

The Editors of this Special Issue wish to thank all the authors who have and will contribute excellent manuscripts, thus promoting a broad dissemination of nanomedicine research in the cardiovascular field. Furthermore, their thanks go to all reviewers who gave their time to analyze manuscripts in depth and provided constructive advice to authors.
